# Supplementary material for: ChatGPT as a Tool for Biostatisticians: A Tutorial on Applications, Opportunities, and Limitations
Source: Stat Med. 2025 Oct 23;44(23-24):e70263. doi: 10.1002/sim.70263 (PMC12548020; doi:10.1002/sim.70263)
Supplement: Supplementary file 1 — Data S1. Supporting Information. [file SIM-44-0-s001.zip › Supplementary Materials Additional.zip!/3.7_Translation_Between_Multiple_Statistical_Programming_Languages/3.7.2_SPSS_to_R/3.7.2_Translation_Chat3.pdf]

R 3

Clear memory

I don't actually have memory on in this chat, so there's nothing for me to clear. If you ever want me to remember something long-term, you can turn memory on in **Settings > Personalization > Memory**.

Want help managing or checking what I remember if memory gets turned on later?

Take the role of an expert biostatistician for this chat

Absolutely — I'm now in expert biostatistician mode. Whether you're working on clinical trial design, survival analysis, generalized linear models, longitudinal data, or anything else stats-heavy in the life sciences, I've got you covered.

What are we diving into today?

I am running the following analysis in SPSS:
DATASET ACTIVATE DataSet5. LOGISTIC REGRESSION VARIABLES V2 /METHOD=FSTEP(LR) V3 V4 V5 V6 V7 V8 V9 V10 V11 V12 V13 V14 V15 V16 V17 V18 V19 V20 V21 V22 V23 V24 V25 V26 V27 V28 V29 V30 V31 V32 /CRITERIA=PIN(0.05) POUT(0.10) ITERATE(20) CUT(0.5).
Translate this into R code.
The data is loaded into R with
temp <- tempfile()
download.file(
"https://archive.ics.uci.edu/static/public/17/breast+cancer+wisconsin+diagnostic.zip",
temp
)
data <- read.table(unz(temp, "wdbc.data"), sep = ",")
data$V2 <- data$V2 == "M"
unlink(temp)

Great — you're running a **forward stepwise logistic regression (based on likelihood ratio tests)** in SPSS, with a binary outcome (`V2`) and a number of predictors (`V3` through `V32`), using PIN = 0.05 and POUT = 0.10 for variable entry and removal.

Here's how to **translate that SPSS logistic regression into R**, using `glm()` and the `step()` function for stepwise selection:

```r
r

# Ensure the outcome is a factor for logistic regression
data$V2 <- factor(data$V2, levels = c(FALSE, TRUE))

# Full model with all predictors
full_model <- glm(V2 ~ ., data = data[, -1], family = binomial)

# Null model (intercept only)
null_model <- glm(V2 ~ 1, data = data[, -1], family = binomial)

# Forward stepwise selection using AIC as default (we'll emulate PIN/POUT next)
stepwise_model <- step(
  null_model,
  scope = list(lower = null_model, upper = full_model),
  direction = "forward",
  trace = 1
)

summary(stepwise_model)
```

Notes:

- `` `data[, -1]` `` removes `` `V1` ``, which is just the ID in the original UCI dataset.
- `` `step()` `` in R uses **AIC** by default, not likelihood-ratio p-values like SPSS does with PIN/POUT.

Optional: Emulating PIN/POUT behavior

For closer control to PIN/POUT, you'd use `` `add1()` `` and `` `drop1()` `` manually in a custom stepwise function, testing variable inclusion/exclusion via **likelihood ratio tests** (i.e., using `` `anova(..., test = "Chisq")` ``).

Want me to build a custom stepwise function that mirrors PIN = 0.05 / POUT = 0.10 more precisely?

Yes

Perfect — here's a custom **forward stepwise logistic regression function in R** that emulates **SPSS's FSTEP(LR)** behavior using likelihood ratio tests with specified **PIN = 0.05** and **POUT = 0.10**.

✅ Custom Stepwise Function (Forward Selection with LR test)

```r
r

custom_forward_stepwise <- function(data, outcome, predictors, pin = 0.05, pout = 0.10, verbose = TI
  # Initial setup
  included <- character(0)
  remaining <- predictors
  formula_base <- as.formula(paste(outcome, "~ 1"))
  model <- glm(formula_base, data = data, family = binomial)

  repeat {
    # Try adding each of the remaining variables and calculate p-value from LR test
    add_tests <- lapply(remaining, function(var) {
      formula_try <- as.formula(paste(outcome, "~", paste(c(included, var), collapse = "+")))
      model_try <- glm(formula_try, data = data, family = binomial)
      pval <- anova(model, model_try, test = "Chisq")$`Pr(>Chi)`[2]
      return(data.frame(variable = var, pval = pval))
    })
```

```
    # Combine into one data frame
    add_results <- do.call(rbind, add_tests)

    # Find the variable with the smallest p-value for entry
    best_candidate <- add_results[which.min(add_results$pval), ]

    if (verbose) {
      cat("Evaluating variable for inclusion:\n")
      print(add_results)
    }

    # Check if it meets the inclusion threshold
    if (best_candidate$pval < pin) {
      included <- c(included, best_candidate$variable)
      remaining <- setdiff(remaining, best_candidate$variable)
      model <- glm(as.formula(paste(outcome, "~", paste(included, collapse = "+"))), data = data, fa
      if (verbose) cat("Added:", best_candidate$variable, "\n\n")
    } else {
      if (verbose) cat("No more variables meet inclusion criteria.\n\n")
      break
    }

    # Check for any variables that now fail the exclusion threshold (POUT)
    if (length(included) > 1) {
      drop_tests <- lapply(included, function(var) {
        reduced <- setdiff(included, var)
        model_reduced <- glm(as.formula(paste(outcome, "~", paste(reduced, collapse = "+"))), data =
        pval <- anova(model_reduced, model, test = "Chisq")$`Pr(>Chi)`[2]
        return(data.frame(variable = var, pval = pval))
      })
      drop_results <- do.call(rbind, drop_tests)

      if (verbose) {
        cat("Evaluating variables for exclusion:\n")
        print(drop_results)
      }

      # Drop any that exceed POUT
      to_remove <- drop_results$variable[drop_results$pval > pout]
      if (length(to_remove) > 0) {
        included <- setdiff(included, to_remove)
        remaining <- union(remaining, to_remove)
        model <- glm(as.formula(paste(outcome, "~", paste(included, collapse = "+"))), data = data,
        if (verbose) cat("Removed:", paste(to_remove, collapse = ", "), "\n\n")
      }
    }
  }

  return(model)
}
```

🧪 Example Usage with Your Data

```
r

# Your setup
data$V2 <- factor(data$V2, levels = c(FALSE, TRUE))
predictors <- paste0("V", 3:32)

# Run custom stepwise LR
final_model <- custom_forward_stepwise(
  data = data[, -1],  # Remove ID column
  outcome = "V2",
  predictors = predictors,
  pin = 0.05,
  pout = 0.10
)
```

```
# Check final model
summary(final_model)
```

This function mirrors SPSS’s **FSTEP(LR)**: it tests variables for entry using **likelihood ratio tests ($p < 0.05$)** and also drops those that become non-significant above **$p > 0.10$**.

Let me know if you want a **backward** version or **bidirectional** (like SPSS’s "stepwise" mode).
